# Disruptions of Sustained Spatial Attention Can Be Resistant to the Distractor’s Prior Reward Associations

**DOI:** 10.3389/fnhum.2021.666731

**Published:** 2021-07-30

**Authors:** Matthew D. Bachman, Madison N. Hunter, Scott A. Huettel, Marty G. Woldorff

**Affiliations:** ^1^Center for Cognitive Neuroscience, Duke University, Durham, NC, United States; ^2^Department of Psychology & Neuroscience, Duke University, Durham, NC, United States; ^3^Department of Psychiatry & Behavioral Sciences, Duke University, Durham, NC, United States

**Keywords:** value-driven attention capture, distraction, sustained spatial attention, SSVEP, reward associations, EEG, RSVP

## Abstract

Attention can be involuntarily biased toward reward-associated distractors (value-driven attentional capture, VDAC). Yet past work has primarily demonstrated this distraction phenomenon during a particular set of circumstances: transient attentional orienting to potentially relevant stimuli occurring in our visual environment. Consequently, it is not well-understood if reward-based attentional capture can occur under other circumstances, such as during sustained visuospatial attention. Using EEG, we investigated whether associating transient distractors with reward value would increase their distractibility and lead to greater decrements in concurrent sustained spatial attention directed elsewhere. Human participants learned to associate three differently colored, laterally presented squares with rewards of varying magnitude (zero, small, and large). These colored squares were then periodically reintroduced as distractors at the same lateral locations during a demanding sustained-attention rapid-serial-visual-presentation (RSVP) task at the midline. Behavioral and neural evidence indicated that participants had successfully learned and maintained the reward associations to the distractors. During the RSVP task, consistent with prior work, we found that the distractors generated dips in the instantaneous amplitude of the steady-state visual evoked potentials (SSVEPs) elicited by the midline RSVP stimuli, indicating that the distractors were indeed transiently disrupting sustained spatial attention. Contrary to our hypotheses, however, the magnitude of this dip did not differ by the magnitude of the distractor’s reward associations. These results indicate that while sustained spatial attention can be impaired by the introduction of distractors at another location, the main distraction process is resistant to the distractors’ reward associations, thus providing evidence of an important boundary condition to value-driven attentional capture.

## Introduction

A burgeoning body of work has documented how attention can be involuntarily drawn to reward-associated stimuli – a phenomenon known as value-driven attentional capture (VDAC; Anderson et al., 2011b; Chelazzi et al., 2013; Failing and Theeuwes, 2018). The biasing of attention associated with VDAC seems to be involuntary and non-strategic; consequently, its ability to help or hinder performance depends upon the task-relevance of the reward-associated stimulus. VDAC can arise from a remarkably broad set of reward-stimulus associations. For example, it has been found that simple elements such as color (Hickey et al., 2010), shapes (Della Libera and Chelazzi, 2009), spatial locations (Chelazzi et al., 2014; Anderson and Kim, 2018), and even complex stimuli such as object categories or scenes (Failing and Theeuwes, 2015; Hickey et al., 2015; Hickey and Peelen, 2017) can be associated with reward.

Once reward-associations have been established, VDAC effects can persist well after reward delivery is no longer occurring. In studies that separate a reward-learning phase and a subsequent test phase that had no rewards, VDAC has been reported at time scales both very short (minutes; Anderson et al., 2011a,b) and quite long (days; Della Libera and Chelazzi, 2009; months; Anderson and Yantis, 2013). Lastly, VDAC is not limited to the visual system, as new reports suggest that it can also occur within the auditory system (Anderson, 2016; Asutay and Västfjäll, 2016; Folyi et al., 2016; Sanz et al., 2018; Folyi and Wentura, 2019) and olfactory system (Pool et al., 2014). This research collectively suggests that VDAC may be a ubiquitous phenomenon that broadly affects many aspects of attentional orienting and behavior – similar to what has been previously reported for attentional capture to physical salience (Hickey et al., 2010). These parallels have generated considerable debate as to whether VDAC modulates the neural mechanisms of attention similarly to other forms of attentional capture (Awh et al., 2012; Anderson, 2019; Bachman et al., 2020).

It has been proposed that attention can be subdivided into several subsystems, such as those governing alerting/sustained vigilance, those governing attentional orienting, and those governing executive control (Petersen and Posner, 2012). VDAC research to-date has predominantly demonstrated the influence of reward-related attentional capture upon attentional orienting, while its influence upon the other two systems is not as well-understood (but see the following behavioral studies: Raymond and O’Brien, 2009; Rutherford et al., 2010; Failing and Theeuwes, 2015; Le Pelley et al., 2017, 2019). Here, we investigated whether VDAC can influence another subsystem of attention: sustained spatial attention/vigilance.

Reward-related attentional capture has been likened to the attentional capture born from the physical salience of a stimulus, which has been shown to be capable of capturing attention during a sustained spatial attention task (Demeter et al., 2008, 2011; Demeter and Woldorff, 2016). Moreover, paradigms that introduce rewards during sustained spatial attention simulate important, real-life circumstances in which one might encounter reward-associated stimuli that could be distracting. For example, advertisements are specifically designed to utilize reward-associated stimuli (e.g., piles of cash or highly valued foods) in order to grab your attention - even when consumers are rarely actually seeking these advertisements out. This competition for attention in the real-world can also have many serious consequences. Although a highway billboard that managed to draw your attention would be considered a “success” by an advertiser, it also disrupts your sustained spatial attention to driving conditions and consequently increases the risk of an accident (Gitelman et al., 2019). Although drivers are usually able to maintain road safety when their attention is distracted for only a brief period of time, longer disruptions make them significantly more likely to become involved in a crash (Klauer et al., 2006). Drivers are also sensitive to the content of a billboard, and are more distracted when the content is more emotional/arousing (Chan and Singhal, 2013, 2015; Chan et al., 2016). Given these and other real-world demonstrations where reward associations can have persistent negative effects on sustained attention and vigilance, we investigated the extent to which associating task-irrelevant stimuli with rewards would increase their overall distractibility during a sustained spatial selective attention task.

We adopted an experimental approach that not only invoked sustained spatial attention via a rapid-serial visual presentation task (RSVP; Demeter and Woldorff, 2016) but also provided a marker of deviations from successful maintenance of attention: decrements in the instantaneous amplitude of the steady-state visual evoked potential (SSVEP) response being elicited by the RSVP stimuli. The SSVEP is a oscillatory neural response that is driven by an ongoing steady-state train of stimuli (Norcia et al., 2015), such as in an RSVP task. Critically, SSVEP amplitude scales in response to attention, increasing when attention is directed toward the eliciting train of stimuli (Müller et al., 1998, 2006) and decreasing when attention is directed elsewhere (Andersen and Müller, 2010; Demeter and Woldorff, 2016). Consequently the SSVEP holds several particular advantages over behavioral measures of sustained spatial attention and distraction. Unlike behavioral measures, it can be measured continuously, regardless of whether a particular stimulus requires a behavioral response. Furthermore, the instantaneous amplitude (InstAmp) of the SSVEP can be assessed independently of event-related potentials (ERPs) triggered by stimuli occurring outside of the RSVP (Demeter and Woldorff, 2016). Thus, measuring the InstAmp of the SSVEP provides a particularly sensitive and robust measurement of changes in sustained spatial attention – allowing us to characterize how previously learned reward associations might disrupt sustained spatial attention.

In sum, prior work has shown that reward-associated distractors can impair attention during visual search. The goal of this particular study was to test for reward-related distraction during sustained spatial attention. We hypothesized that task-irrelevant distractor stimuli that had been previously associated with larger rewards would lead to increased impairments in sustained spatial attention, as measured by the InstAmp of the oscillatory activity at the SSVEP frequency. To achieve this goal, we adapted the original RSVP task utilized by Demeter and Woldorff (2016), but with the task-irrelevant distractors here being colored squares that had been previously associated with different levels of reward, to see if those reward associations impacted the distraction effects. In particular, participants were first introduced to these colored squares as targets in a reward oddball task. In this other task, participants learned that each colored square was tied to varying amounts of money, thus creating different reward associations for each stimulus when these squares were reencountered as distractors in the later RSVP task. In addition, we wanted to make sure that our participants not only established reward associations for each stimulus type, but also that they maintained them over the course of the experiment (rather than their being extinguished once they became irrelevant in the RSVP task). To this end, we had our participants refresh these reward associations by periodically having them complete additional blocks of the reward-oddball task that were interleaved in between sets of blocks of the RSVP task. Thus, our design and our neural measures of sustained spatial attention allowed us to understand the degree to which distractor reward associations disrupted sustained spatial attention to the RSVP task.

## Materials and Methods

### Participants

Our sample consisted of 23 participants drawn from the Duke University and Durham, NC, communities. Data from one participant was dropped from this initial sample due to technical issues during data collection that led to substantial EEG data loss. Data from another participant was removed because EEG artifacts (mainly eye movements and eye blinks) were occurring in over half of their epoched SSVEP data. Thus, our final dataset comprised 21 participants (Age: 25.0 ± 2.8 years; 11 females). Participants were compensated by either course credit or via a base payment of $15/hour. In addition to this compensation, participants could win more money for their performance during the reward oddball task (see details below; average bonus: $7.67 ± $0.47). All participants had normal color vision (Ishihara Test for Color Blindness; Ishihara, 1917) and had normal or corrected-to-normal visual acuity. Participants gave their written consent before starting the study in accordance with a protocol approved by the Duke Institutional Review Board.

### Overall Experimental Design

**FIGURE 1:**
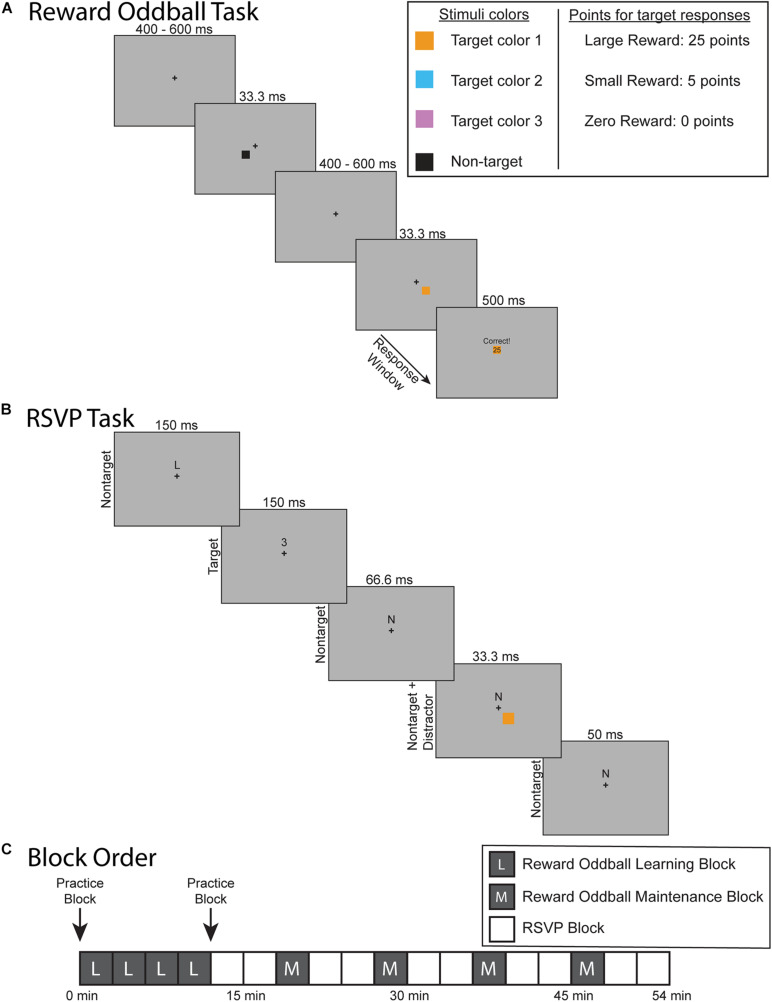
Experiment and protocol design. **(A)** Reward oddball task. Participants responded to the location of colored, lateralized target. Accurate and sufficiently fast responses would be rewarded with points that were later converted into a monetary bonus. **(B)** The rapid serial visual presentation (RSVP) task. Participants fixated on a cross at the center of the screen while covertly attending to a stream of letter and number characters slightly above fixation – and pressing a button whenever a number character appeared (8% of the character stimuli). Periodically during this task, one of the lateralized colored squares would reappear on the screen as a reward-associated distractor. **(C)** Overview of the experimental protocol. The experiment began with four blocks of the reward oddball task so that participants learned to associate each colored square with different levels of reward. They then completed the RSVP task to test how these reward-associated distractors impacted sustained spatial attention. The RSVP task blocks were then interleaved with blocks of the reward oddball task in order to maintain these reward associations.

The experimental protocol was composed of two separate tasks: a reward oddball task (Figure 1A) and an RSVP task that elicited steady-state visual-evoked potentials (SSVEPs) (Figure 1B). The goal of the reward oddball task was to associate lateralized squares of different colors with reward associations of varying magnitude. These lateralized colored squares would later be reintroduced during the midline RSVP task as transient, reward-associated distractors. Participants completed four blocks of the reward oddball task at the start of the experimental session, and then performed additional blocks of that task interspersed throughout the remainder of the experiment. This structure was designed to ensure that the participants would not only form reward-associations to each colored square type, but would also maintain them over the course of the experiment (instead of the learned response being extinguished over the session). In total, participants completed 8 blocks of the reward oddball task and 10 blocks of the RSVP task (Figure 1C). Each block was roughly 3 min long, and the entire experiment took just under an hour to complete, including breaks. Both experimental tasks were presented using the Presentation 21.0 software program (Neurobehavioral Systems, Inc., Berkeley, CA), set to a 60 Hz frame rate. Participants responded using a Logitech gamepad held in both hands.

### Reward Oddball Task

In the reward oddball task, participants responded to infrequent target stimuli that appeared unpredictably within a stream of non-target stimuli (Mullette-Gillman et al., 2011). Participants fixated upon a center of the screen across the entire task. Each trial began with just the central fixation cross. After a 400–600 ms delay a square would also appear either below and to the left or below and to the right of the fixation cross. This square would remain on the screen for 33.3 ms and consist of one of four possible colors. On most trials (70%) the square would be colored black, indicating that it was a non-target square to which the participant should not respond. Participants were told that squares of any of the other three colors (orange, pink, and cyan) were target squares and that they were to respond to the target square’s spatial location (left or right) by pressing the corresponding left or right button on the gamepad with their left or right index finger, respectively. The three target colors were selected via pilot testing to have equivalent physical salience. This was achieved by adjusting the lightness of all colors until they were perceived as having the same level of luminance in a flicker-fusion paradigm (Simonson and Brozek, 1952). This procedure equates the global sensory input energy level for each target color, removing potential sensory differences that could affect the ERPs (Donohue et al., 2016; Harris et al., 2016; Bachman et al., 2020). Each target color was equally likely to appear on each trial (10% for each target color), and targets were equally likely to appear on the left or right side. Each block of the reward oddball task consisted of 126 trials.

The critical element of the reward oddball task was the yoking of variable reward-associations to each of these three target colors. Participants were told that accurate and sufficiently quick responses to the target squares would result in a variable number of points – to be later converted to a monetary bonus ($1 for every 350 points) – that differed for each target color. One target color was associated with 25 points (i.e., large value), another with 5 points (i.e., small value), and one with 0 points (i.e., zero value). The specific association of each color (orange, cyan, and pink) to each of the potential reward levels (large, small, and zero) was counterbalanced across subjects. In order to push our participants to remain highly motivated during the experiment we required them to accurately respond within a certain response window in order to receive their points. This response window was individually tailored to the participant’s last five response times for that target type, titrated to keep them at an 80% accuracy rate. After a response was made or the response window elapsed, there was a small delay of 300–500 ms before a feedback screen appeared for 500 ms. If participants responded quickly enough within the response window, then the feedback screen would tell them that they were “Correct,” along with how many points they had won. A copy of the target square was also displayed in the middle of the feedback screen. If the participants were unable to respond within the response window then the screen would display “Slow” and awarded them 0 points. Incorrect responses were also awarded 0 points and were displayed with the message “Incorrect.”

### RSVP Task

The RSVP task was adapted from the distraction task used in Demeter and Woldorff (2016). During this task participants remained fixated upon a central fixation cross while covertly attending to a stream of characters presented just above fixation, which were mostly letters with infrequent numbers, which changed every 150 ms (i.e., at a 6.7 Hz rate). They were instructed to respond to the infrequently occurring number-stimulus targets (8% of characters) using their right thumb to press a button on the gamepad. They were also told that the lateralized colored squares from the reward-oddball task would be periodically reappearing during this task, but that they were irrelevant in this task and should be ignored. These colored squares were presented in the same manner as in the reward-oddball task, but here now as transient, irrelevant distractors. More specifically, we maintained the same stimulus properties as used in the reward oddball task: the squares appeared in the same spatial locations, lasted for the same period of time, and were similarly counterbalanced by color (orange, cyan, and pink) and side (left or right). However, we note that the duration of the distractors (33.3 ms) was briefer than the duration of each character stimulus (150 ms) in the RSVP stream, and thus they could appear on any frame during the presentation of the RSVP stimulus (e.g., simultaneously with the RSVP stimulus onset, in the middle of the RSVP stimulus, etc.). Distractors appeared every 600–1050 ms, with uniform probability over that interval. Participants were also told that no money could be earned during the RSVP task; thus, these distractors could not produce any rewards during the RSVP task. The black, non-target squares were never used in the RSVP task. Each block of the RSVP task (3 min) consisted of 1179 SSVEP stimuli, with concurrent presentation of ∼214 of the lateralized colored-square distractors.

### EEG Acquisition and Preprocessing

Participants were seated 60 cm away from a 24-inch monitor in a dimly lit, sound-attenuated, and electrically shielded room. They were fit with an elastic EEG cap equipped with 64 active Ag/AgCl electrodes (actiCAP; Brain Vision LLC, Morrisville, NC). The caps were custom designed for extended coverage of the full head, with equally spaced electrodes and coverage stretching from slightly above the eyebrows to below the inion (Woldorff et al., 2002). The electrode impedances were kept below 15 kΩ and were referenced to the right mastoid during recording. The EEG signal was recorded using a three-staged cascaded integrator-comb filter with a corner frequency of 130 Hz and using a sampling rate of 500 Hz per channel (actiCHamp; Brain Vision LLC, Morrisville, NC). Eye movements were monitored using vertical and horizontal EOG channels in conjunction with a closed-circuit zoom-lens camera.

Offline processing of the data was performed using the EEGLAB Toolbox (Delorme and Makeig, 2004). First, a 30-Hz non-causal low-pass filter was applied to the data. The data were then downsampled to 250 Hz, followed by application of a 0.01 Hz high-pass non-causal filter. All data were rereferenced to the algebraic average of the two mastoids. Channels for which the electrode had lost good connectivity to the scalp during the recording or were excessively noisy for any other reasons were excluded from each individual subject’s dataset before applying independent component analysis (ICA) to correct for eye blinks and eye movements. After this correction, these previously removed noisy channels were included back into the dataset using a spherical-spline interpolation solution. Epochs of the data were taken around the onset of the different stimuli in each task. For the reward oddball task, epochs of −400 to 900 ms were taken around the onset of each target and non-target stimulus. In the RSVP task, epochs of −600 to 1000 ms were taken around the onset of every stimulus (targets, non-targets, and distractors). All of these epochs were baseline corrected from −200 to 0 ms. We then identified and removed epochs that still contained ocular artifacts using two functions that identified rapid changes in EEG amplitude associated with muscular contractions. The first step function was used to identify remaining horizontal eye movements in each epoch while the second step function identified any remaining blink artifacts. Finally, we identified and removed epochs in which activity in any channel passed ±100 μV.

### Reward Oddball Analyses

For the reward-oddball task, the behavioral analyses focused on the response times (RTs) between the onset of a target and the participant’s response. We analyzed the data from the first four reward-association-learning oddball blocks and from the reward-association-maintenance oddball blocks separately.

For the ERP analyses, we assessed the amplitude of the target-evoked P300 wave for neural evidence that our participants had learned to associate different reward levels with each of the target types. We focused on the P300 because it has a robust literature demonstrating its sensitivity to reward, namely that its amplitude is larger for larger reward magnitudes (Sato et al., 2005; Bernat et al., 2015; Bachman et al., 2020). Consequently, we hypothesized that, if the reward associations to each target had been established, then we would see an increase in P300 amplitude with higher reward level. In this dataset, we defined the P300 as the mean amplitude of activity occurring between 300 and 600 ms, averaged over the electrodes Pz and POz. As with the behavioral data, separate averages were extracted for the learning and maintenance blocks.

### SSVEP Analyses

Our behavioral measures focused upon target-detection performance when a distractor occurred in close proximity to a target. We measured the temporal proximity of the onset of each distractor relative to the onset of a numeric target in the RSVP stream (Timing Offset), limiting our selection to distractors that occurred from −450 to +150 ms around the RSVP target onset. This time range corresponded to the time period of the presentation of the three RSVP characters preceding a target, in addition to the one occurring during the presentation of the target itself. We assessed this impact of this variable upon both target reaction time and target accuracy. Accuracy was defined in the same manner as Demeter and Woldorff (2016), where a response was marked as “accurate” if it was made 150 to 1200 ms after the onset of a target in the RSVP stream.

We estimated instantaneous amplitude (InstAmp) of the SSVEP response using procedures similar to prior studies (e.g., Gladwin et al., 2006; Demeter and Woldorff, 2016). More specifically, the EEG data were convolved using a complex Morlet wavelet set to the frequency of the SSVEP response (6.7 Hz), with a standard deviation of 2 Hz in the frequency domain. We focused on the SSVEP at electrode sites POz, O1, and O2, the same cluster of occipital-parietal electrodes used in Demeter and Woldorff (2016). We initially compared the InstAmp during periods when there was no distractor (i.e., when there was no distractor occurring with the non-target stimulus or in the 450 ms preceding it) to the InstAmp during periods containing a transient distractor. We recognized that the introduction of a distractor stimulus will evoke an EEG response (i.e., an ERP) that contains some power at the SSVEP’s driving frequency, irrespective of any effects upon sustained spatial attention and the RSVP-driven SSVEPs. To remove this potential confound, we used the procedures of Demeter and Woldorff (2016). First, we extracted every epoch for each distractor at each reward-level; the average ERPs of these epochs did not contain any signal related to the SSVEP because they were equally likely to begin at any screen frame of the RSVP’s character-stimulus presentation (0, 16, 33, 50, 66, 83, 100, 116, and 133 ms). Consequently, any activity from the steady-state stream was averaged out during the distractor-ERP averaging, leaving the ERP to the distractors. The next step extracted the InstAmp across time at the RSVP frequency from each of these ERPs, as a means of calculating how much confounding power was being introduced by the transient distractor event. Third, the resulting ERP-related InstAmp activity was convolved across a 9-point delta function, corresponding to each possible point along which a distractor could appear during the presentation of a character stimulus. Finally, this convolved signal was then subtracted from the InstAmp time course extracted from non-target + distractor trials, thereby removing contribution from the distractor ERP-related InstAmp and leaving a clean representation of the non-target InstAmp, but when a distractor had been present.

### Statistical Analysis

All of our behavioral measures were submitted to linear mixed-effect models (i.e., fixed effects within subject, random effects across subjects) through MATLAB’s fitglme function. For the reward oddball task, we extracted and analyzed response times but not accuracy, since our task was designed to keep each participant’s accuracy at 80% for each target. For the RSVP task we extracted target accuracy and target response time. The fixed-effect factors used in the reward oddball mixed-effect models were Block (Learning Blocks vs Maintenance Blocks) and Reward Level (Large, Small, and Zero), both of which were set as categorical variables. As a result, both Block and Reward Level were calculated relative to their “baseline” variables of the Learning Blocks or the Zero-Reward targets, respectively. For the SSVEPs, the fixed effects were composed of target Offset (i.e., the onset of the distractor from −450 to +150 ms relative to the target onset), as well as Reward Level, which was again calculated as a categorical variable.

Our neural measures were submitted to repeated-measures analyses of variance (rmANOVAs) using SPSS 26 (IBM Corp. Released 2019. IBM SPSS, Armonk, NY, United States). Significance for each rmANOVA was defined as *p* < 0.05. The significance of main effects was determined following Bonferroni-corrected pairwise comparisons. Significant interaction terms were assessed using Bonferroni-corrected paired sample *t*-tests. We also conducted several *post-hoc* Bayesian rmANOVAs to test the strength of evidence in support of the null hypothesis. We conducted these rmANOVAs through JASP using default priors with a Cauchy distribution (Rouder et al., 2012) and in comparison to the null model. The priors specifically used an r scale fixed-effects value of 0.5, an r scale random-effects value of 1, and an r scale covariate value of 0.354.

## Results

### Learning and Maintaining Reward Associations in the Reward Oddball Task

**FIGURE 2:**
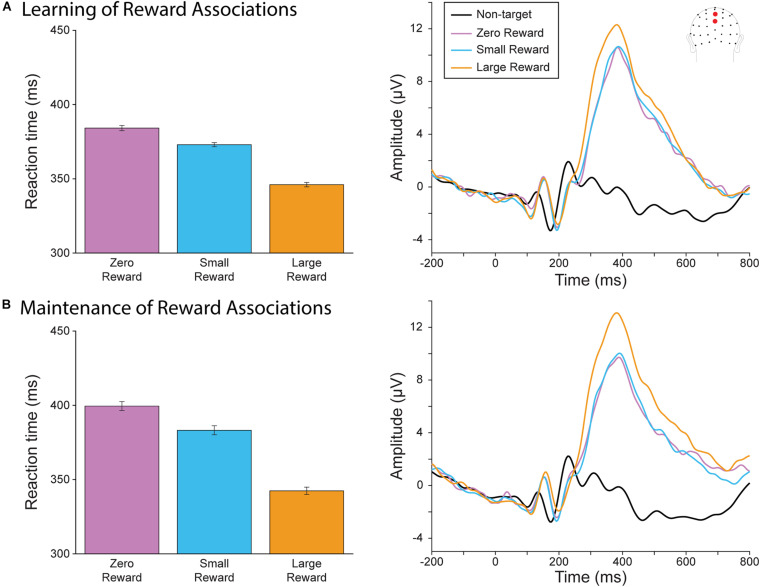
Behavioral and neural results from the reward oddball task. **(A)** Reaction time (*left*) and P300 (*right)* during the learning blocks. Participants responded faster for large-reward targets, which also evoked a larger P300. **(B)** Reaction time (*left*) and P300 (*right*) during the later, maintenance blocks of the reward oddball task. Similar to the results in the earlier blocks, participants responded faster and had a larger P300 for large-reward targets.

We first assessed whether participants had learned to associate each target square color with its corresponding reward magnitude during the learning blocks (Figure 2A) and maintained these associations throughout the rest of the experiment (Figure 2B). We evaluated reaction time by fitting a linear mixed effect model of reaction time fitted with the categorical variable of Reward Level (Large vs. Zero and Small vs. Zero) and Block (Maintenance vs Learning). First, we found that both large and small reward targets resulted in faster response times than zero reward targets (*R*^2^ = 0.61; Large vs. Zero Reward slope = slope = −38.10, *t*(9066) = −10.37, *p* < 0.0001; Small vs Zero Reward slope = −11.19, *t(*9066) = −3.04, *p* = 0.002), indicating that our participants were performing better when the reward incentive was present. We also found a significant effect of Block, where responses became slower during the later blocks (Maintenance vs Learning slope = 15.35, *t*(9066) = 4.17, *p* < 0.001). This likely reflects the fixed order of these blocks, where participants may have been more fatigued during the later Maintenance blocks. There was a significant interaction between Block and the Large vs Zero term, indicating that the difference between these categories became even larger during the maintenance block (Maintenance vs Learning × Large vs Zero Reward slope = −19.00, *t*(9066) = −3.66, *p* < 0.0001). However, there was no significant interaction between Block and Small vs Zero (Small vs. Zero slope = −5.11, *t*(9066) = −0.98, *p* = 0.326). Thus, our participants were not only able to learn these reward associations, but also to maintain them (and even increase their relative differences) after they had become irrelevant during the sustained spatial attention task.

We also assessed the P300 amplitude during the reward-oddball blocks for neural evidence that they had associated each target color with their corresponding degrees of reward, and whether they were able to maintain these associations throughout the experiment. A 3 × 2 rmANOVA of Reward Level (Large, Small, and Zero) by Block (Training vs Maintenance) found a significant main effect of target reward magnitude (*F*(2,40) = 18.37, *p* < 0.001). As we hypothesized, P300 amplitude was significant for large-reward targets compared to small-reward (Δ 1.82 μV, *p* < 0.001) and zero-reward targets (Δ 2.06 μV, *p* = 0.001). There was no significant difference between small- and zero-reward targets (Δ 0.24 μV, *p* = 1.000). There was no significant main effect of block (*F*(1,20) = 0.009, *p* = 0.926), and unlike our behavioral results, the interaction effect did not reach significance (*F*(2,40) = 2.73, *p* = 0.077). Thus, neural evidence also supports the conclusion that reward associations to each stimulus-color type were learned and maintained throughout the experiment.

### RSVP Behavioral Results

**FIGURE 3:**
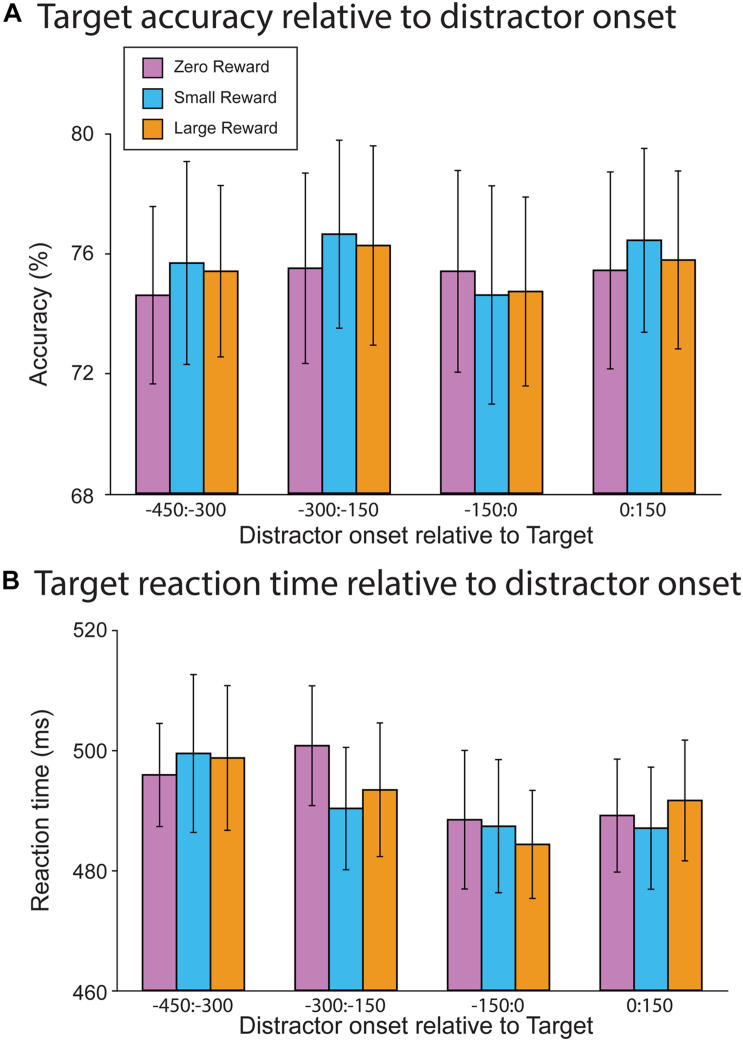
Behavioral results from the RSVP task. **(A)** Target accuracy as a function of relative distractor onset time. Distractors did not impact target accuracy as a function of either onset time or reward magnitude. **(B)** Target reaction time as a function of distractor onset time. There was a trend toward faster reaction times when the distractor occurred just prior to target onset. However, this did not differ as a function of reward magnitude.

We first attempted to replicate the results of Demeter and Woldorff (2016), who observed that distractors impacted target accuracy when they occurred during the presentation of a target, but had no effect upon response time. Accordingly, we assessed how target accuracy (Figure 3A) and response time (Figure 3B) depended on the distractor onset (from −450 ms pre-target to 150 ms post-target). Contrary to both our hypothesis and prior work, we did not find a significant association between accuracy and distractor offset (*R*^2^ = 0.08, Offset slope = −0.00, *t*(13745) = 1.11, *p* = 0.267). This initial model was followed by a second model that incorporated the reward-level terms; in it, neither the Large vs. Zero nor Small vs. Zero comparison resulted in a significant main effect (*R*^2^ = 0.08, Large vs. Zero slope = 0.08, *t*(13745) = 1.02, *p* = 0.31; Small vs. Zero slope = 0.035, *t*(13745) = 0.46, *p* = 0.646). Additionally, neither of these terms significantly interacted with distractor offset (*R*^2^ = 0.08, Large vs. Zero × Offset slope = 0.00, *t*(13745) = 0.39, *p* = 0.698; Small vs. Zero × Offset slope = −0.00, *t*(13745) = −0.16, *p* = 0.877). Thus, neither distractor offset nor reward-level had a significant impact upon target accuracy.

In contrast, RTs did vary as a function of distractor offset, whereby distractors occurring closer to the onset of a target resulted in faster responses (*R*^2^ = 0.05, Offset slope = −0.03, *t*(11246) = −3.15, *p* = 0.002). A mixed-effect model incorporating reward level did not find a main effect for either term (*R*^2^ = 0.05, Large vs. Zero slope = −2.55, *t*(11248) = −0.56, *p* = 0.575; Small vs. Zero slope = −0.61, *t*(11248) = −1.33, *p* = 0.894). Furthermore, neither of these reward-levels interacted with distractor offset (Large vs. Zero × Offset slope = 0.02, *t*(11248) = 0.79, *p* = 0.428; Small vs. Zero × Offset slope = −0.00, *t*(11248) = −0.18, *p* = 0.855). So while distractors did generally decrease participants’ response times, this effect was not sensitive to the reward associations of each particular distractor.

### SSVEP InstAmp Results

**FIGURE 4:**
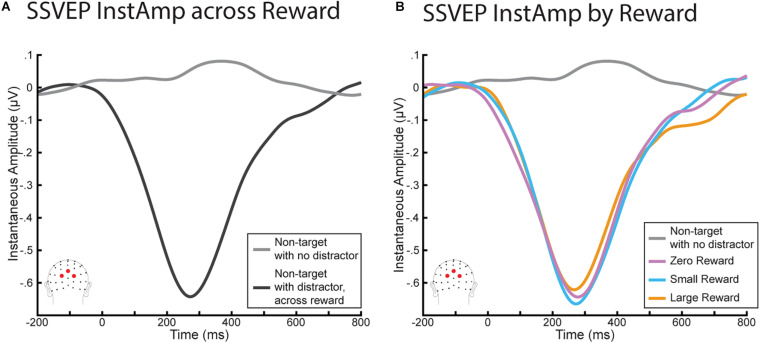
InstAmp results from the RSVP task. **(A)** Comparing InstAmp when non-targets were presented with and without a distractor of any reward magnitude. Distractors elicited a significant decrease in InstAmp from 0 to 600 ms. **(B)** InstAmp for distractors by reward. While all distractors generated a significant decrease in InstAmp over this period, this decrease did not differ significantly as a function of reward magnitude.

We again began with a similar analysis as in Demeter and Woldorff (2016), who found a significant decrease in InstAmp that began with the onset of the distractor and did not return to baseline until ∼600 ms later. As we were also interested in understanding the time course of the InstAmp, we conducted a parallel set of analyses (Figure 4A) using a 2 (stimulus: non-target with no distractor vs non-target with distractor) × 6 (time bin: −150:750 in 150 ms increments) rmANOVA. This rmANOVA yielded a main effect of stimulus (*F*(1,20) = 45.23, *p* < 0.001), a main effect of time bin (*F*(5,100) = 28.34, *p* < 0.001), and a significant interaction term (*F*(5,100) = 39.38, *p* < 0.001). Paired sample *t*-tests found that while InstAmps to each non-target were not significantly different before the onset of the distractor (*t*(20) = 1.59, *p* = 0.774), they began to diverge shortly after the distractor appeared. This resulted in a significant decrease in InstAmp for the non-target with distractors (*t*(20) = 6.29, *p* < 0.001). This decrease peaked just before 300 m but remained significantly lower in every time bin (0:150, 150:300, 300:450, and 450:650; all *p*’s < 0.001) until the 600 to 750 ms bin (*t*(20) = 1.46, *p* = 0.966). Thus, these InstAmp results replicate those reported in Demeter and Woldorff (2016).

We then assessed how this decrease in InstAmp differed as a function of the distractor’s reward associations (Figure 4B). In particular, in order to understand how the time course of the InstAmp was affected by each distractor’s reward associations, we conducted a 3 (stimulus: large-reward distractor, small-reward distractor, zero-reward distractor) × 6 (time bins) rmANOVA. There was once again a significant main effect of time bin (*F*(50,100) = 36.78, *p* < 0.001), encapsulating how the amplitude of the InstAmp dipped across time. However, there was no main effect of reward (*F*(2,40) = 0.007, *p* = 0.993) or interaction between reward and time bin (*F*(10,200) = 1.33, *p* = 0.216). Visual inspection of the data suggested that there might be a late effect of reward between 600 and 800 ms, whereby the InstAmp following a high-reward stimulus may have returned to baseline more slowly (i.e., stay decreased longer) than for small- and zero- reward distractors. We tested this possibility via a *post-hoc* 3 × 1 rmANOVA of mean InstAmp in this time range by each distractor type, but did not find a significant effect of reward in this late window (*F*(2,40) = 2.55, *p* = 0.091).

Our finding that a distractor’s reward associations had a non-significant influence upon the SSVEP InstAmp ran directly counter to our primary hypotheses, but because these analyses were based on frequentist statistics we were limited in our ability to make conclusions as to whether this finding actually supported the null-hypothesis. To provide evidence that reward-associations had no influence upon this disruption of sustained spatial attention, we conducted several additional *post-hoc* Bayesian rmANOVAs and interpreted the resulting Bayes Factors. Bayes Factors can show evidence for either the alternative or null hypothesis, with values over 1 indicating evidence supporting the alternative hypothesis and values under 1 indicating evidence supporting the null hypothesis. The strength of that evidence is indicated by how much it deviates from 1. We chose to focus on two particular analyses and interpreted the resulting Bayes Factor using the terminology that is commonly employed in that work (e.g., Wetzels et al., 2011). First, we assessed the InstAmp measure around the lowest point of the InstAmp dip (i.e., reflecting the peak of the attentional distraction from the main task) by averaging the data from 150 to 450 ms separately for each distractor type and submitting it to a 1 × 3 Bayesian rmANOVA. The results of this model suggested substantial evidence for the null hypothesis in this time period (*BF*_10_ = 0.17). In light of the trend toward a late effect of reward in the 600–800 ms time period, we also performed a Bayes analysis in that time period. Submitting this data to a 1 × 3 Bayesian rmANOVA indicated only weak (often termed “anecdotal,” e.g., Wetzels et al., 2011) evidence for the null hypothesis (*BF*_10_ = 0.84) that this later time period was also resistant to reward associations of the distractor. In sum, we found that that the initial, main decrease in the InstAmp of the relevant task resulting from the occurrence of a distracting stimulus was resistant to the distractors’ reward associations. While our analyses of the later InstAmp time period also suggested resistance to the distractor’s reward associations, our results could not provide conclusive evidence in this regard.

## Discussion

Previous research has shown how reward-associated stimuli can involuntarily capture attention during an attentional orienting task, but it is not well understood how this phenomenon extends to other forms of attention. In this study, we examined whether VDAC would still occur when participants were selectively sustaining focused attention to another task in another spatial location. We hypothesized that distractors associated with larger rewards, even when occurring in a different location, would capture more attention and thus lead to greater disruptions in participants’ sustained spatial attention. Although the physical introduction of a transient distractor did indeed decrease sustained spatial attention, as reflected by a dip in the SSVEP response elicited by the RSVP task, this disruption was not significantly affected by the distractors’ reward associations (i.e., the disruption did not differ between zero-, small-, and large-rewarding distractor stimuli). This indicates that the reward associations of the distractor stimuli did not have a significant effect on sustained spatial attention beyond the distraction associated with the physical introduction of a new and irrelevant stimulus.

Although we did not find support for our primary hypothesis, namely that the reward magnitude of the distractor stimuli would alter the SSVEP response to the relevant RSVP task, it is notable that the introduction of a distractor still resulted in a disruption of sustained spatial attention, aligning with both our expectations and prior empirical work (Demeter and Woldorff, 2016). Our results thus speak to the ongoing debate on whether visual salience and reward salience exert similar or different influences upon attention. Early reports of VDAC noted that it generated similar behavioral outcomes as objects with physical salience (Hickey et al., 2010), leading to theories that they have similar influences upon attentional resources (Anderson, 2019). However, other theoretical perspectives have suggested that these two types of salience (i.e., physical and value-driven) must rely upon different neural mechanisms, as physical salience is borne from a stimulus’s physical characteristics while value-driven salience forms after learning from previously rewarding experiences (Awh et al., 2012). Empirical evidence has been building toward this latter perspective, as several studies have shown that physical and value-driven salience contribute unique variance to the degree of behavioral distraction (Anderson et al., 2011a; Wang et al., 2013; Gong and Liu, 2018). In addition, our group has recently provided neural evidence supporting this view, finding that physical salience affects the *speed* of attentional orienting, whereas value-driven salience affects the *strength* of attentional orienting (Bachman et al., 2020). The idea that reward-associated distraction utilizes different mechanisms relative to the physical salience of distractors aligns with our main findings that the disruptions of sustained spatial attention can be resistant to distractor reward associations. Moreover, this also suggests another key difference between reward-related salience and physical salience: that VDAC does not exert a universal influence upon all forms of attention like physical salience does, but rather that influence is stronger in some forms of attention and weaker or perhaps non-existent in others. Although the current set of findings is limited in its strength in speaking directly on this point, we posit that they do support the growing consensus that reward-associated distraction derives from unique neural mechanisms.

Our results differ from the handful of previous behavioral studies investigating the influence of VDAC on non-spatial attention that did find modulation by distractor-reward value (Raymond and O’Brien, 2009; Rutherford et al., 2010; Failing and Theeuwes, 2015; Le Pelley et al., 2017, 2019). There are some notable parallels between these studies and ours, as well as one key distinction. As in the current experiment, participants in these other studies learned to associate different stimuli with reward value in a learning phase before completing an RSVP task. These reward-associated stimuli were later introduced as irrelevant distractor stimuli just before a target was presented in an attentionally demanding RSVP task. However, a critical difference between these other studies and ours is that their reward-associated distractors occurred directly within the relevant visual stream (i.e., where spatial attention was focused) while ours occurred outside of it. Thus, these prior studies used a task structure more akin to an attentional blink paradigm (Raymond et al., 1992) and assessed how these reward-associated stimuli within an attended stream can take up attentional resources, while our study focused more upon how reward-associated stimuli occurring in an unattended spatial location might involuntarily capture spatial attention. This distinction may identify an important boundary condition on the occurrence of VDAC.

On a similar note, there are a few other papers showing certain circumstances where lateralized reward-associated distractors can pull attention away from targets in a visual search array (Wang et al., 2014, 2015). The targets in these studies were notable in that they always appeared in a single midline position; thus, participants could have sustained their attention toward this known target location in anticipation of the onset of the array. Despite this, participants were still distracted when the distractor was close to the target and previously associated with large rewards. Similar evidence has also been shown in the auditory domain, where participants attended more to distracting tones played in a task-irrelevant channel when they were previously associated with rewards (Folyi and Wentura, 2019). One potential reason why these other studies found evidence of reward-related distraction while ours did not may derive from the different perceptual demands of the various tasks. In our experiment, participants needed to sustain their spatial attention to a continuous, rapidly changing stream of changing stimuli, a process which likely induced a higher perceptual load relative to the tasks in these other studies. This factor might be relevant because perceptual load can modulate a participant’s susceptibility for distraction, with larger perceptual loads decreasing the perceptual processing of irrelevant stimuli from other locations and the propensity of being distracted by them (Lavie, 2005, 2010; Elliott and Giesbrecht, 2015). Consequently, it may be that sustained spatial attention can be resistant to reward-related modulation of distraction when a participant’s perceptual load to a main task is high, but is less so when the load is low, but future work will be needed to confirm this conclusion.

Another possible requirement for VDAC is that the reward-associated stimulus may need to share a defining feature with a target. This idea is taken from the contingent attentional capture theory (Folk et al., 1992), which has frequently demonstrated this principle using unrewarded distractors in sustained spatial attention tasks (Folk et al., 2002, 2008; Leblanc et al., 2008; Moore and Weissman, 2010). For example, Serences et al. (2005) demonstrated how an unrewarded distractor in a peripheral, explicitly irrelevant location could disrupt sustained spatial attention, but in particular when it shared the same color as a target in the sustained attention task. Thus, our results may have been different had our distractors been lateralized colored numbers instead of squares, or had the targets in our midline RSVP task been defined as stimuli of particular colors that matched those used for the reward-associated distractors. Such correspondence may also play a role in some of the most common forms of VDAC visual search tasks where target and non-target stimuli share several features (e.g., Anderson et al., 2011b); future work will be needed to assess the validity of this possibility.

We designed our experimental protocol to rule out one potentially confounding factor, namely that participants might have extinguished their learned response to the reward-associated stimuli over time when they were no longer relevant in the RSVP task. To this end, we interleaved additional blocks of the reward-oddball task throughout the experimental session, ensuring that our participants regularly refreshed their reward associations. The fact that both response times and the P300s in these later maintenance blocks fit our predicted pattern of effects regarding reward value suggests that this design was successful. Moreover, several pieces of evidence suggest that VDAC is a relatively stable, persistent phenomenon. For example, many VDAC studies consist of only one learning phase and never refresh these associations and yet are able to find robust effects (e.g., Anderson et al., 2011a). A few studies have even found that VDAC effects even when an extended period of time has passed between the learning and test phases, on the time course of days (Della Libera and Chelazzi, 2009; Chelazzi et al., 2014) and even months (Anderson and Yantis, 2013). Of course, these reward associations are not impervious to change, but studies indicating such changes in the reward associations tend to use paradigms where the reward-associations continue to change and require active, continual learning of new and updated reward associations (Oemisch et al., 2017), which was not a factor in our experiment. Together, these results suggest that our primary finding, that distractor-reward associations did not lead to additional decrements in sustained spatial attention, was not due to a change in the underlying learned stimulus-reward association.

Identifying potential boundary conditions such as reported here may help to us fully characterize VDAC and understand the extent to which it can impact attention and cognition. Similar efforts have been underway in the domain of face processing; for example, emotional expressions may, under certain conditions, have a limited effect on early processing and attentional biasing (Schindler and Bublatzky, 2020). The findings of the current study may prove useful for a similar effort in the domain of reward-association history.

There are several important limitations to our study that bear consideration. First, we found no behavioral evidence of distraction on target accuracy, even though Demeter and Woldorff (2016) had previously found such an effect. This difference may have been the product of several critical differences between our experimental protocols. In particular, Demeter and Woldorff alternated between two different sustained spatial attention tasks (i.e., one with distractors and one without distractors), which ultimately required their participants to sustain their attention for an entire hour. Continually engaging in sustained attention is taxing and can lead to general decrements in vigilance and performance (Warm et al., 2008), which may have made participants more vulnerable to distraction. Conversely, our experiment included intermittent blocks of the reward-oddball task that increased participation engagement (as seen both in raw performance measures and in verbal reports from participants after the experiment). This increased engagement may have counteracted the likelihood of a vigilance decrement and made the participants less susceptible to distraction. Nonetheless, the sensitivity of our InstAmp neural measure ensured that our assay of sustained spatial attention was not constrained to behavior alone, allowing us to have additional sensitivity for capturing possible distraction by reward. Another limitation of this study was our sample size (*n* = 21). While we slightly exceeded the number of subjects used in Demeter and Woldorff, a larger dataset may have been able to provide more conclusive evidence of our findings. Although through our Bayes analysis we were able to provide substantial evidence that the peak of the InstAmp dip was not sensitive to reward-associations, we were able to provide only weak evidence for such insensitivity in the late period of the InstAmp. While the corresponding Bayes factor (0.84) slightly favored the null hypothesis, it was also very close to the pivot point (1.0), at which there is not really sufficient evidence in either direction. Accordingly, our data does not provide a firm conclusion as to whether this later time period is or is not sensitive to reward association history. Future work with a larger sample size would be of value to replicate these findings and confirm that there is indeed no effect of reward at the peak of the attentional distraction (i.e., at the peak of the SSVEP InstAmp dip), and to more fully assess whether or not there could be a later effect. Finally, we note that the time-window used to assess the P300 in the reward-oddball task also overlaps with the average response times, and that targets with higher rewards led to both faster RTs and larger P300s. It is possible that these different motor processes may have influenced these P300 differences above-and-beyond the pure influence of reward magnitude. While we cannot rule out this possibility, it is known that the P300 exhibits differences in reward even in tasks that require no motor responses (e.g., Yeung et al., 2005). In addition, the motor-related neural activity related to the manual responses would have been more frontal in their spatial distribution. Our P300’s were taken from a posterior distribution, suggesting that the effects on our measures of the P300 were not likely due to manual response activity. Regardless, the results would still reflect the general conclusion that our participants had successfully learned and maintained the different reward associations to the different colored squares.

As noted in the introduction, our RSVP task simulates real-life scenarios in which an actor must balance sustained attentional demands against infrequent reward-associated distractions – as when drivers encounters a highway billboard along their journeys. While our initial hypotheses of reward-association modulating the distraction effects were not supported by these findings, upon closer inspection they would seem to dovetail with a number of studies investigating this real-world phenomenon. Although billboards do indeed draw attention away from the road and negatively impact driving behavior (Edquist et al., 2011; Meuleners et al., 2020), they do not account for the majority of accidents. Studies have shown that most distraction-related accidents come from events or activity occurring within the vehicle, while external distractions such as billboards only account for 6–9% of distraction-related traffic crashes (Wallace, 2003; Young et al., 2007). Additionally, drivers are less susceptible to distraction when driving requires a high perceptual load (Young et al., 2007; Marciano and Yeshurun, 2012; cf. Hoekstra-Atwood et al., 2017), a cognitive condition that would seem to be the case under the focused spatial attention circumstances of the current study. The conjunction of these studies of real-world driving accidents, along with our findings, provides converging evidence that sustained, strongly focused, spatial attention in both real and simulated circumstances is relatively robust against the impact of value-driven distraction from other spatial locations.

In summary, our study replicates prior findings showing that a concurrent distractor stimulus appearing in a different location than a sustained visual attention task can impair the focus of that spatial attention. However, our findings indicate that the level of this attentional impairment was, at least in our sample, resistant to the distractor’s reward associations. Evidence for this resistance was strong at the initial, main, distraction process, but very weak later on in time, such that that we cannot rule out the possibility that the reward-association history of a distractor could influence sustained spatial attention after some time delay. Accordingly, we do not suggest that sustained spatial attention is completely impervious to the effects of distractor reward-associations, but that there may be boundaries to the extent to which VDAC can influence the capture of attention. Our findings, in conjunction with prior work, has identified several possibilities for such boundary conditions and/or contexts that constrain the ability for reward associations to influence sustained spatial attention. Clarifying the nature of these conditions and context and timing will refine our understanding of the VDAC phenomenon, and thus illuminate when, how, and the extent to which it plays a role in cognition and behavior.

## Data Availability Statement

The raw data supporting the conclusions of this article will be made available by the authors upon request.

## Ethics Statement

The studies involving human participants were reviewed and approved by the Duke Institutional Review Board. All participants provided their written informed consent to participate in this study.

## Author Contributions

MB, SH, and MW conceived and designed the study. The data were collected and analyzed by MB and MH under the supervision of MW and SH. MB wrote the initial draft of the manuscript and it was edited by SH and MW. The manuscript was read and approved by all authors.

## Conflict of Interest

The authors declare that the research was conducted in the absence of any commercial or financial relationships that could be construed as a potential conflict of interest.

## Publisher’s Note

All claims expressed in this article are solely those of the authors and do not necessarily represent those of their affiliated organizations, or those of the publisher, the editors and the reviewers. Any product that may be evaluated in this article, or claim that may be made by its manufacturer, is not guaranteed or endorsed by the publisher.
